# An integrative knowledge graph for rare diseases, derived from the Genetic and Rare Diseases Information Center (GARD)

**DOI:** 10.1186/s13326-020-00232-y

**Published:** 2020-11-12

**Authors:** Qian Zhu, Dac-Trung Nguyen, Ivan Grishagin, Noel Southall, Eric Sid, Anne Pariser

**Affiliations:** 1 Division of Pre-Clinical Innovation, National Center for Advancing Translational Sciences (NCATS), National Institutes of Health (NIH), Rockville, MD 20850 USA; 2 Office of Rare Disease Research, National Center for Advancing Translational Sciences (NCATS), National Institutes of Health (NIH), Bethesda, MD 20892 USA

**Keywords:** GARD, Rare diseases, Ontology, Data integration, Knowledge graph

## Abstract

**Background:**

The Genetic and Rare Diseases (GARD) Information Center was established by the National Institutes of Health (NIH) to provide freely accessible consumer health information on over 6500 genetic and rare diseases. As the cumulative scientific understanding and underlying evidence for these diseases have expanded over time, existing practices to generate knowledge from these publications and resources have not been able to keep pace. Through determining the applicability of computational approaches to enhance or replace manual curation tasks, we aim to both improve the sustainability and relevance of consumer health information, but also to develop a foundational database, from which translational science researchers may start to unravel disease characteristics that are vital to the research process.

**Results:**

We developed a meta-ontology based integrative knowledge graph for rare diseases in Neo4j. This integrative knowledge graph includes a total of 3,819,623 nodes and 84,223,681 relations from 34 different biomedical data resources, including curated drug and rare disease associations. Semi-automatic mappings were generated for 2154 unique FDA orphan designations to 776 unique GARD diseases, and 3322 unique FDA designated drugs to UNII, as well as 180,363 associations between drug and indication from Inxight Drugs, which were integrated into the knowledge graph. We conducted four case studies to demonstrate the capabilities of this integrative knowledge graph in accelerating the curation of scientific understanding on rare diseases through the generation of disease mappings/profiles and pathogenesis associations.

**Conclusions:**

By integrating well-established database resources, we developed an integrative knowledge graph containing a large volume of biomedical and research data. Demonstration of several immediate use cases and limitations of this process reveal both the potential feasibility and barriers of utilizing graph-based resources and approaches to support their use by providers of consumer health information, such as GARD, that may struggle with the needs of maintaining knowledge reliant on an evolving and growing evidence-base. Finally, the successful integration of these datasets into a freely accessible knowledge graph highlights an opportunity to take a translational science view on the field of rare diseases by enabling researchers to identify disease characteristics, which may play a role in the translation of discover across different research domains.

## Introduction

An estimated 30 million people in the United States are affected by a rare disease, which is defined as a disease that affects fewer than 200,000 individuals in the United States [1]. The majority of rare disease are thought to have a genetic etiology [2] with studies reporting them responsible for almost 10% of adult and 30% of pediatric hospitalizations [3]. Despite the great heterogeneity of diseases included in this definition, many patients and their families share in common struggles, such as with diagnostic delay leading to “an average of 7.6 years” from initial onset of symptoms to receiving a diagnosis and requiring the involvement of 7.3 physicians on average [4]. These shared challenges faced in the broader rare disease patient community are often due to a lack of either up-to-date information or awareness amongst providers and the public at large. Efforts to tackle these issues led to the passage of the Rare Disease Act of 2002 and the establishment of several programs by the National Institutes of Health (NIH) to improve research activities and public access to information on rare diseases. In particular, the Genetic and Rare Diseases (GARD) information center was charged with providing freely accessible consumer health information in plain language, and it has been investigating the challenge of shifting from an entirely manual process to leveraging computational approaches to curate the accumulated biomedical and clinical research knowledge of over 6500 rare diseases, and more rapidly make information accessible 1) to educate patients, families, and health care providers with more accurate and real-time knowledge about a rare disease, and 2) to support novel scientific research efforts and apply disease-agnostic translational science approaches to the field of rare diseases as a whole [5].

Given the pace of ongoing scientific discovery, parsing through the accumulated research publications and conveying this knowledge in a plain language format accessible to low-health literacy audiences presents a significant task for a single disease, let alone for over 6500 rare diseases. Thus, a huge amount of effort to accumulate and curate data for rare diseases has been made globally. For instance, the GARD Information Center provides interpretable profiles in plain language for each rare disease [5]; Orphanet focuses on expert manual curation of a disease’s clinical presentation [6]; and Online Mendelian Inheritance in Man® (OMIM®) conducts a similar expert-driven focus on defining genotype and phenotype relationships [7]. The discreteness of such heterogeneous data, however, impedes their direct use for consumer audiences. To overcome this barrier, in this study, we integrated these well-known resources in one knowledge graph to semantically interconnect all data together by means of the data points as nodes and their relationships as edges, as a first step in bridging the use of these resources in consumer-facing health information.

Biomedical data integration is an important and technical approach to tackling biomedical problems. Current progress in computational technology allows vast data storages and powerful computational processes to be more affordable and accessible. As a result, biomedical scientists have gradually gained an awareness of the importance of pooling diverse types of data pertaining to a specific medical entity to enhance their research understanding [8]. Representing integrative data in the form of a graph has attracted many interests, particularly in the biomedical domain. Karczewski K, et al. have reviewed and discussed the potential and usage and challenges of integrating diverse types of omics data for human health and disease [9]. Biomedical Informatics Research Network (BIRN) is an integrative resource by semantically integrating data produced by multiple institutions for data analysis on Neurosciences [10]. Similar efforts have also begun to emerge with applications directed at the field in rare disease, such as the semantic Diseasecard, which integrates rare disease data from distinct sources in a semantic web environment [11]. A similar EU platform, RD-Connect connects databases, registries, biobanks and clinical bioinformatics to support research in discovering new genes, biomarkers, and therapeutic targets more quickly and efficiently [12]. The Monarch Initiative as another analytic platform, semantically integrates genotype and phenotype data across differing species and sources [13], and has led to the establishment of MONDO (Monarch Merged Disease Ontology) [14] as a cohesive ontology for connecting many of the disease databases and resources. The integrative knowledge graph we introduce in this study applies well-established rare disease data drawn from GARD, Orphanet, OMIM and MONDO as a backbone, and then expands to a wide spectrum of additional biomedical data, including phenotypes, genes and curated FDA approved drugs and FDA orphan drug designations.

There are demonstrated merits and successes in using graph database to support the management of large biomedical datasets. While relational databases excel at managing relationships between data, graph databases provide unique abilities to manage n-th degree relationships among complex types of biomedical data. Furthermore, graph databases are particularly apt at representing hierarchical data, such as disease categories and complex semantic relationships among different types of data. Neo4j as a graph database management system [15], has been widely applied in such use cases within the biomedical domain. Such as, Gratzl S, et al. demonstrated the utility of Neo4j in developing integrated visual analysis platform for biomedical data [16]; Himmelstein D, et al. constructed Hetionet, an integrative Neo4j network that encodes knowledge from millions of biomedical studies to prioritize drugs for repurposing [17]. In this paper, we introduce this rare diseases integrative knowledge graph, built in Neo4j as a backend graph database ingesting a large variety of biomedical datasets. We detail data preparation and entity resolution methodologies in generating initial insights and results, and the potential benefits for utilizing a knowledge graph-based approach to interpret biomedical research at a scale and pace that would be unsustainable when limited to the manual curation efforts that define current processes used in curating consumer health information.

## Materials

At the time of writing, the knowledge graph integrates 34 different biomedical datasets including GARD. We briefly describe several primary resources as below.

### Rare disease related data resources

Besides GARD data retrieved from our internal database, all other datasets were downloaded from NCBO Bioportal [18].

**GARD** is currently managed by the Office of Rare Diseases Research (ORDR) within the National Center for Advancing Translational Sciences (NCATS), has remained an important portal for patients, health-care professionals, and researchers seeking to understand the current state of genetic and rare diseases. GARD includes curated disease information comprised of 15 different sections, such as summary, diagnosis, inheritance, etc. Notably, not all of GARD diseases have a complete list of these 15 information sections, due to data unavailability at the time of curation and update. In this study, we extracted and applied disease specific information sections, if applicable from our internal GARD database. Other sections, such as Resources, Organizations will be explored in the future [5].

**Orphanet** is a unique resource, gathering and improving knowledge on rare diseases so as to improve the diagnosis, care and treatment of patients with rare diseases [6].

**Monarch Disease Ontology (MONDO)** is a semi-automatically constructed ontology that merges multiple disease resources to yield a coherent merged ontology [14].

**Online Mendelian Inheritance in Man® (OMIM®)** is a comprehensive, authoritative compendium of human genes and genetic phenotypes. The full-text, referenced overviews in OMIM contain information on all known mendelian disorders and over 15,000 genes [7].

**Human Phenotype Ontology (HPO)** provides a standardized vocabulary of phenotypic abnormalities encountered in human disease [19].

### FDA orphan drugs

**FDA orphan drug designation** provides orphan designations to drugs and biologics, which are defined as those intended for the safe and effective treatment, diagnosis or prevention of rare diseases/disorders [20]. In this study, we employed orphan drug designation data from the FDA [21], several examples of FDA orphan drug designations retrieved from the FDA are shown in Table 1. Specifically we utilized the associations between FDA designated drugs (the column of “Generic Name” in Table 1) and their designations (the column of “Orphan Designation” in Table 1). Although the data is presented in a structured form, orphan designation is captured in free text, such as examples shown in Table 1. In that manner, additional curation was conducted in this study to be able to map orphan designations to GARD diseases and designated drugs to UNII (Unique Ingredient Identifier).

**Table 1:** Examples of FDA orphan drug designations

#	Generic Name	Orphan Designation	Designation Date	Designation Status
1	((1r, 4r)-N1-(2-benzyl-7-(2-methyl-2H-tetrazol-5-yl)-9H-pyrimido[4,5-b]indol-4-yl)cyclohexane-1,4-diamine dihydrobromide dihydrate)-Expanded cord blood	Prevention of Graft-versus-Host-Disease	12/13/2018	Designated
2	((4-(3-benzyl-4-hydroxybenzyl)-3,5-dimethylphenoxy)methyl)phosphonic acid	Treatment of X-linked adrenoleukodystrophy	12/05/2016	Designated
3	(+)-(2S)-2-(4-chloro-2-methoxyphenyl)-2-{[3-methoxy-5-(methylsulfonyl)phenyl]amino}-1-[5-(trifluoromethoxy)-1H-indol-3-yl]ethanone	Treatment of dengue virus infection	12/26/2017	Designated

**Inxight Drugs** is a drug resource developed by NCATS. Inxight Drugs [22] incorporates the most comprehensive subset of substances and related biological mechanisms pertaining to translational research and connects them to the appropriate disease indications. As part of Inxight Drugs, explicit connections between drugs and conditions were manually identified from scientific articles, press releases, FDA labels, and large-scale databases (e.g. AdisInsight [23]). For those identified associations, the curators manually matched conditions to MeSH, Disease Ontology (DO), and drugs to UNII (Unique Ingredient Identifier). For example, one association presenting in Inxight Drugs is as “CYROMAZINE” (with UNII: CA49Y29RA9) has indication of “MYIASIS, CUTANEOUS MYIASIS OF SHEEP”. In this study, we extracted associations between FDA approved drugs and diseases, and integrated them into our integrative knowledge graph.

## Methods

In this paper, we detail the process of developing the integrative knowledge graph for rare diseases with inclusion of multiple well-known biomedical datasets including GARD. We also demonstrate the use of this integrative graph to support biomedical research for rare diseases. More details about this process is described as below.

### Data collection

GARD data is curated in two folds, manual curation by information specialists from GARD, and programmatic extraction from Orphanet. The curated data is stored in a relational database, from where we extracted GARD data for this study. GARD provides comprehensive information about rare diseases from different aspects, including summary, sign and symptoms, treatment, organizations, research, resources, references, etc. but only disease related information, including summary, symptoms, prognosis, treatment, etc. for 6323 rare diseases has been applied, given our main focus of this study is rare disease based data integration.

We applied FDA orphan drug designations and FDA approved drugs from Inxight Drugs in this study. FDA orphan drug designations includes associations between designated drugs and orphan designations, but such information is not represented in a structured and standardized form. Several examples of orphan designations are listed in the Material section. Accordingly, we mapped orphan designations to GARD diseases and designated drugs to Unique Ingredient Identifier (UNII), in two steps: we first programmatically mapped GARD diseases to orphan designations via exact text match, i.e., identifying mentions of GARD disease names in orphan designation texts. In order to avoid any missed mappings, we obtained synonyms for each GARD diseases if applicable, and applied the synonym list along with GARD disease names for mapping. Then for those unmapped orphan designations, our curation team led by one co-author, IG, conducted manual mapping between orphan designations and GARD. Meanwhile, the curators manually mapped designated drugs to UNII. In order to track and improve mappings in the future, the curators labeled each mapping as “Done”, “Approximate” or “Failed” to indicate the status of mappings accordingly. Furthermore, they inserted specific comments for reasons of those tags being assigned, particularly for “Approximate” and “Failed”. For instance, one comment of “the designation phrase is incomplete” was inserted for the orphan designation of “Treatment of pediatric patients 0 to”. In the meantime, the curation team extended their previous mappings of FDA approved orphan drugs to UNII for the project of Inxight Drugs, with newly approved orphan drugs by FDA.

The aforementioned rare disease related datasets described in the Materials section, are publicly available via NCBO Bioportal [18], from where we downloaded those datasets in the form of OWL (the Web Ontology Language). Prior to integrate those datasets, data cleanup was performed. For instance, in the OMIM file, OMIM ID has been labelled with prefix of “OMIM” and “MIM”, same as the Orphanet data, Orphanet ID labelled with prefix of “ORPHA” or “ORPHANET”. In these cases, we restricted with “OMIM” and “ORPHA” as prefix only.

### Meta-ontology definition

Based on the nature of the collected data and our research need, we pre-defined a meta-ontology to formally capture and represent semantic relationships among different types of data and guide data integration subsequently.

#### Primary class definition

We collected various types of data, we defined primary classes accordingly, including Condition, Drug, Gene, etc. which are listed in Table 1. Besides “Condition”, we also derived 32 rare disease categories from GARD as individual disease classes, such as “Blood Diseases”, “Endocrine Diseases” and “Parasitic Diseases” [24], to better capture rare disease information precisely. Particularly, one class named “DATA” has been defined as one data container to manage data properties. In addition, we adopted some classes from the original data sets, for instance, a list of UMLS semantic types [25], such as T047 for “Disease or Syndrom”, T028 for “Gene or Genome”, haven been used by multiple resources including MeSH, NCI Thesaurus. In Table 2, we listed primary classes along with their associated data sources.

**Table 2:** Primary classes and the corresponding data sources

Classes	Primary data resources	Abbreviations used in this study
Condition and Designated	• Inxight Drugs • FDA Orphan Drug Designations	• S_RANCHO-DISEASE-DRUG_2018-12-18_13–30 • S_FDAORPHANGARD_20190216
Rare Diseases and 32 different rare disease categories from GARD	• GARD • MONDO • Orphanet • OMIM	• S_GARD • S_MONDO • S_ORDO • S_OMIM
Human Phenotype	• HPO • Phenotype And Trait Ontology • Mammalian Phenotype Ontology	• S_HPO • S_PATO • S_MP
Drug	• Inxight Drugs • FDA Orphan Drug Designations • VA National Drug File (VANDF)^a^ • MeSH^a^	• S_RANCHO-DISEASE-DRUG_2018-12-18_13–30 • S_FDAORPHANGARD_20190216 • S_VANDF • S_MESH
Chemical	• ChEBI • Thesaurus^a^	• S_CHEBI • S_THESAURUS
Gene	• GHR • Ontology of Genes and Genomes • MedGen^a^ • Thesaurus^a^	• S_GHR • S_OGG • S_MEDGEN • S_THESAURUS
Protein	• Protein Ontology	• S_CL • S_CLO • S_MP • S_HP
Cell	• Cell Ontology • Cell Line Ontology • Thesaurus^a^ • MedGen^a^	• S_CL • S_CLO • S_THESAURUS • S_MEDGEN
Tissue	• Thesaurus^a^	• S_THESAURUS
DATA	• All data properties are store in this class	• DATA

^**a**^Semantic types have been adopted to represent different classes from VANDF, MeSH, MedGen, and Thesaurus, such as T109 representing “Organic Chemical”, T121 representing “Pharmacologic Substance”, T025 for “Cell”, T028 for “Gene or Genome”, etc.

#### Object property definition

In order to capture semantic relationships among primary classes, we defined object predicates by two strategies: 1) based on semantic relationships, such as “has_phenotype” was defined to represent relationship between disease concepts (i.e., concepts belonging to the classes of “Condition” or one of 32 rare disease categories) and phenotypes belonging to the class of “Human Phenotype”; “subclassOf” was defined to represent parent-child relationships. 2) To establish new linkages across different resources for data integration, for instance, “N_Name” defined to develop mappings based on concept names and/or their synonyms, “I_Code” defined to develop mappings based on concept codes, including MONDO ID, OMIM ID, etc. “I_Gene” to develop mappings based on Gene symbols. Table 3 shows the main object properties defined for this study.

**Table 3:** Object properties

Object Property	Relationships
has_phenotype	Disease and Phenotype
subClassOf	Parent and Child concepts
equivalentClass	Equivalence (in terms of their class extension) of two named classes.
exactMatch	Two concepts with a high degree of confidence that the concepts can be used interchangeably.
R_rel	Relationships derived from other resources, such as “has_inheritance_type” from the HPO
N_Name	Mappings based on concepts names and/or their synonyms.
I_Code	Mappings based on identifiers, such as UMLSCUI, MONDO ID, HPO ID.
I_GENE	Mappings based on Gene symbols
PAYLOAD	Concept and DATA node

#### Data property definition

Data properties link individual concepts to their data values. While we defined data properties to capture information for each class accordingly, we also adopted data properties from original data resources. For instance, NCI Thesaurus includes a list of annotation properties and object properties, we adopted them if applicable in this study. All data properties are stored in the class of “DATA”. The data properties along with some explanation are shown in Table 4.

**Table 4:** Data properties

Data property	Corresponding class	Explanation
ConditionDoId, ConditionDoValue, ConditionMeshId, ContitionName, ConditionFdaUse, ConditionComment	Condition	• ConditionDoId: Mapped Disease Ontology ID; • ConditionMeshId: Mapped MeSH ID
gard_id, Categories, is_rare, name, synonyms, xrefs, Sign and symptom, Treatment, Diagnosis, etc.	GARD Rare Diseases	• is_rare: An indicator of “RARE” disease; • xrefs: mappings to other resources, including MONDO, Orphanet;
CompoundName, CompoundSmiles, CAS, UNII, OfflabelUseComment	Drug	
ID, Label, URI, IAO_0000115	Gene	• IAO_0000115: Definition of the concept
ID, IAO_0000115, Label, Synonym, uri, Gene Symbol	Protein	• IAO_0000115: Definition of the concept • Id: Protein Ontology identifier
IAO_0000115, hasDbXref, hasRelatedSynonym, label uri	Tissue	• IAO_0000115: Definition of the concept • hasDbXref: external references
hasDbXref, IAO_0000115 id, label, uri	Human Phenotype	• Id: HPO identifier
Annotation properties and object properties are adopted from NCI Thesaurus	Chemical	

### Knowledge graph generation

To construct the knowledge graph, we utilized our in-house data integration framework named stitcher (https://github.com/ncats/stitcher)**.** Data resources in their native formats (e.g., OWL, RDF, JSON, XML, text delimited, etc.) were consumed by stitcher to build this integrated multigraph with all entities as a single Neo4j database. The “stitching” process for each data source was done by mapping the relevant data attributes to a set of well-known stitch keys (e.g., “N_Name”, “I_CODE”). The integrative knowledge graph is publicly accessible at https://disease.ncats.io.

## Results

We generated the rare disease based integrative knowledge graph in Neo4j, with a total number of 3,819,623 nodes and 84,223,681 relations from 34 different biomedical data resources. We summarize results for each step towards the knowledge graph generation as below.

### Results for GARD data collection

We included disease-related information for 6323 GARD unique diseases. Table 5 shows the statistical results of GARD data included in this study. As we mentioned before, not all of GARD diseases include a complete list of information sections, we incorporated disease information sections available at the time of data extraction.

**Table 5:** Statistical results of GARD data

Sections of GARD profile	Number of GARD diseases
Summary	3077
Symptoms	868
Cause	862
Inheritance	729
Diagnosis	615
Treatment	1058
Prognosis	602

### Results for FDA orphan drug designation mapping

Through automated and manual mapping processes, total 2154 unique FDA orphan designations have been successfully mapped to 942 unique GARD diseases, and 3322 unique designated drugs have been mapped to UNII. The mappings were grouped into four categories, three categories based on mapping status from the manual process, “Done”, “Approximate” and “Failed”, plus one category of “Automation” via the programmatic process. The grouped results are shown in Tables 6 and 7. A number of orphan designations were failed to map to GARD and a list of designated drugs cannot be mapped to UNII, we will discuss the failure reasons in the Discussion section.

**Table 6:** Mapping results for FDA orphan drug designations to GARD

Mapping methods		#Mappings	#FDA orphan designations	#GARD diseases
Automation		1449	1162	482
Manual process	Done	1041	859	491
	Approximate	4,92	339	220
	Failed	618	618	NA

**Table 7:** Mapping results for FDA designated Drugs to UNII

Drug mappings	
# Unique designated drugs mapped to UNII	3322
# Unique designated drugs unable to map to UNII	525

### Results for Inxight drugs

Drug and disease associations were extracted from Inxight Drugs and integrated into the graph. The results are listed in the Table 8.

**Table 8:** Statistical results for curated drug-disease-associations from Inxight Drugs

Total number of nodes	12,138
• Number of drugs	8218
• Number of conditions	3920
Total number of relationships	180,363

### Results for the entire knowledge graph

At the time of writing, we collected and integrated 34 different data resources in this integrative knowledge graph. Notably, relevant datasets will be continually integrated into this graph. Table 9 shows a snapshot of the summarized results. More up-to-date results on included datasets can be retrieved by running the Cypher Query 1 from our Neo4j database.

**Table 9:** Statistical results of some primary resources from the knowledge graph

Datasets	Number of Nodes
BRENDA Tissue & Enzyme Source Ontology	6352
Human Phenotype Ontology (HPO)	40,260
Genetics Home Reference (GHR)	1307
National Organization for Rare Disorders (NORD)	1281
Medical Subject Headings (MeSH)	279,463
Monarch Disease Ontology (MONDO)	118,962
Online Mendelian Inheritance in Man (OMIM)	109,624
Orphanet	43,610
Ontology of Genes & Genomes (OGG)	69,973
Chemical Entities of Biological Interest (ChEBI)	134,358
VA National Drug File (VANDF)	28,278
Phenotype And Trait Ontology (PATO)	3504
Inxight Drugs	19,817
FDA Orphan Drug Designations	6074
GARD	6323

**Cypher Query 1:** match (n:DATASOURCE) return n.name, n.instances.

**Description**: searching resource names and the number of nodes for all available resources in our neo4j graph.

## Biomedical applications derived for rare diseases

In order to demonstrate potential use of this knowledge graph, we performed four case studies to support further enhancement of GARD program, in addition to facilitate rare disease research.

### Disease mappings across multiple disease resources

With this integrative knowledge graph, we are able to generate a disease mapping matrix across different resources for directing data harmonization effort. In this case study, we generated disease mappings across ten disease resources based on their concept names/synonyms (via “N_Name”) and/or concept identifiers (via “I_CODE”), which allows us to review the results from two aspects towards data harmonization, 1) the mappings based on “N_Name” can be applied to evaluate the effort on data normalization (i.e., whether concept names from each resource are using standard form); 2) the mappings based on “I_CODE” can be applied to evaluate the effort on data integration (i.e., whether external references from each resource are accurate and complete). One query example to develop such mappings is shown as Cypher Query 2. The complete mapping results are shown in Table 10. Apparently more than half of those resources, including Orphanet, NORD, MeSH, NCI-t, DO and GHR have more precise one-to-one mappings to GARD, comparing to the mappings between MONDO, OMIM, MedGen and GARD, which include more one to many mappings. Furthermore, comparing to the total number of concepts from each resource, the numbers of mapped concepts are quite small, which illustrates that, 1) there is a need to propose a global data standard to represent rare disease data in a standardized form; 2) more data harmonization effort is needed to have more complete mappings.

**Table 10:** Disease mappings across multiple disease resources

	GARD	Orphanet	MONDO	OMIM	NORD	MeSH	NCI-t^**a**^	DO^**b**^	GHR	MedGen
**GARD**	**6073**	4198	5698	3481	1166	3914	1442	2920	860	4733
**Orphanet**	4260	**38,573**	8670	4475	672	3618	1476	3619	628	6726
**MONDO**	6613	11,612	**23,560**	8954	1683	8697	3549	11,678	1262	14,652
**OMIM**	4824	7445	11,655	**13,605**	1089	8530	2771	6575	869	13,599
**NORD**	1065	649	1190	725	**1281**	959	647	826	382	1070
**MeSH**	3836	3328	7935	5872	1027	**8937**	2147	3526	860	8935
**NCI-t**	1506	1471	3483	1722	728	2523	**5487**	2588	509	4642
**DO**	2925	4403	9987	4358	915	3771	2401	**13,210**	737	6782
**GHR**	852	628	1138	566	383	899	499	736	**1308**	1069
**MedGen**	6600	10,717	18,004	11,067	1678	12,239	5041	10,503	1251	**52,448**

^a^ NCI-t: NCI Thesaurus; ^b^ Disease Ontology

**Cypher Query 2:** match (a:DATA)-- > (n:S_GARD)-[:N_Name|:I_CODE*1]-(m:S_OMIM:T047^1^) < −-(b:DATA) where a.is_rare = true return count(distinct n) as S_GARD, count(distinct m) as S_OMIM.

**Description**: searching for the number of mappings between GARD and OMIM based on concept name/code by restricting OMIM disease concepts and GARD rare diseases only.

### Disease profile generation

**Fig. 1:**
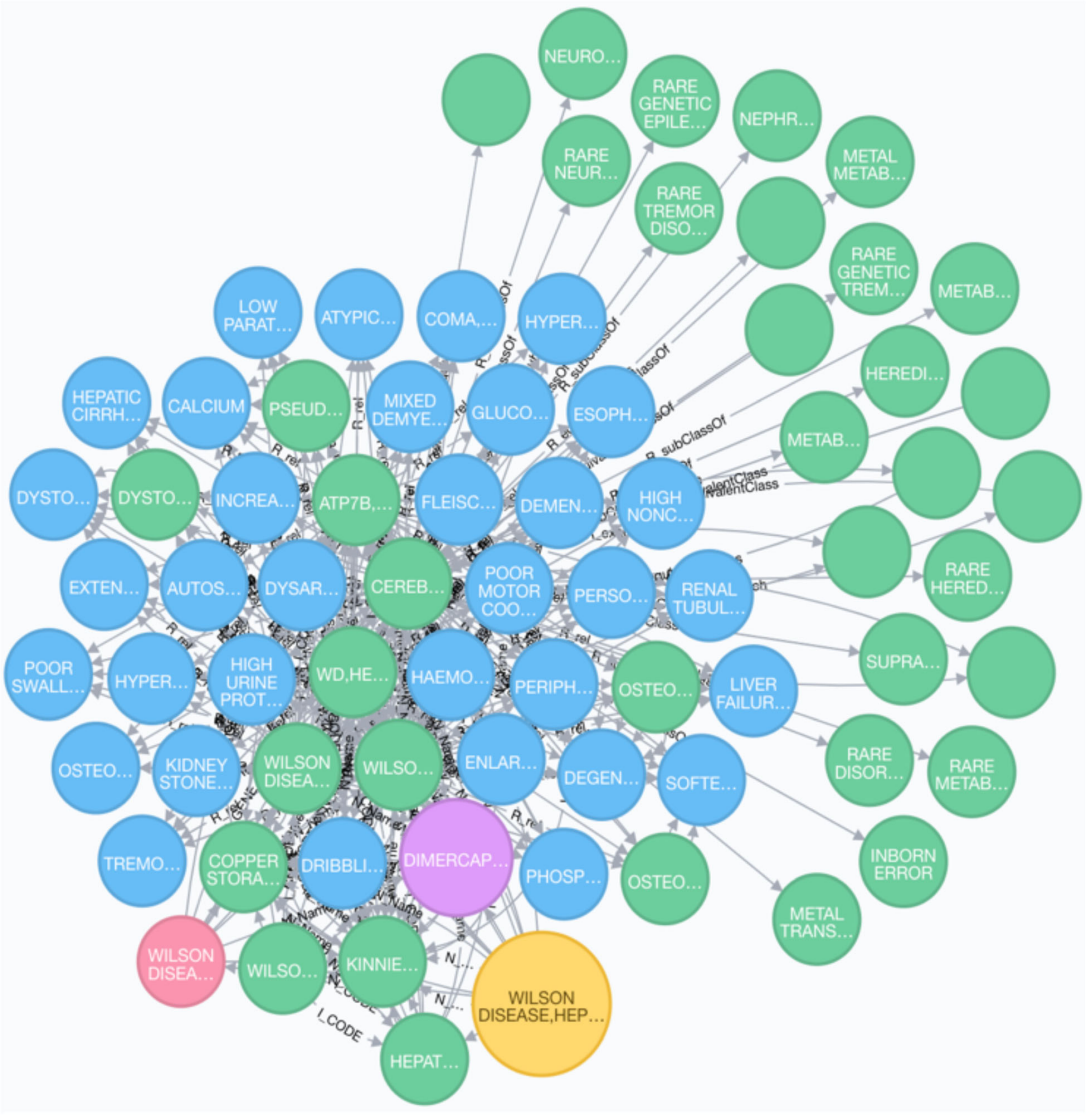
Disease profile for “WILSON DISEASE” (large yellow nodes denotes GARD diseases; blue nodes denotes phenotypes; purple nodes denotes drugs; red nodes denotes genes; green nodes denotes mappings to other resources)

Disease profile, particularly for rare diseases, can be an incredible resource to support scientific research and clinical decision-making. The integrative knowledge graph introduced in this manuscript includes diverse types of data, such as diseases, drugs, genes, proteins, etc. which can be applied to generate disease profiles. Figure 1 shows one disease profile generated for “WILSON DISEASE”. The profile was comprised of detailed disease information, i.e., summary, cause, inheritance from GARD, and associated genes, phenotypes, drugs, etc. from other integrated data resources. The graph shown in the Fig. 1a was generated by searching nodes that are at most three layers away from the query node of “WILSON DISEASE”. The node color indicates different classes of the nodes. Figure 1b shows detailed information contained in the nodes that mapped to “WILSON DISEASE”, which is represented as a single large yellow node at the base.

### Data harmonization rule generation for GARD

**Fig. 2:**
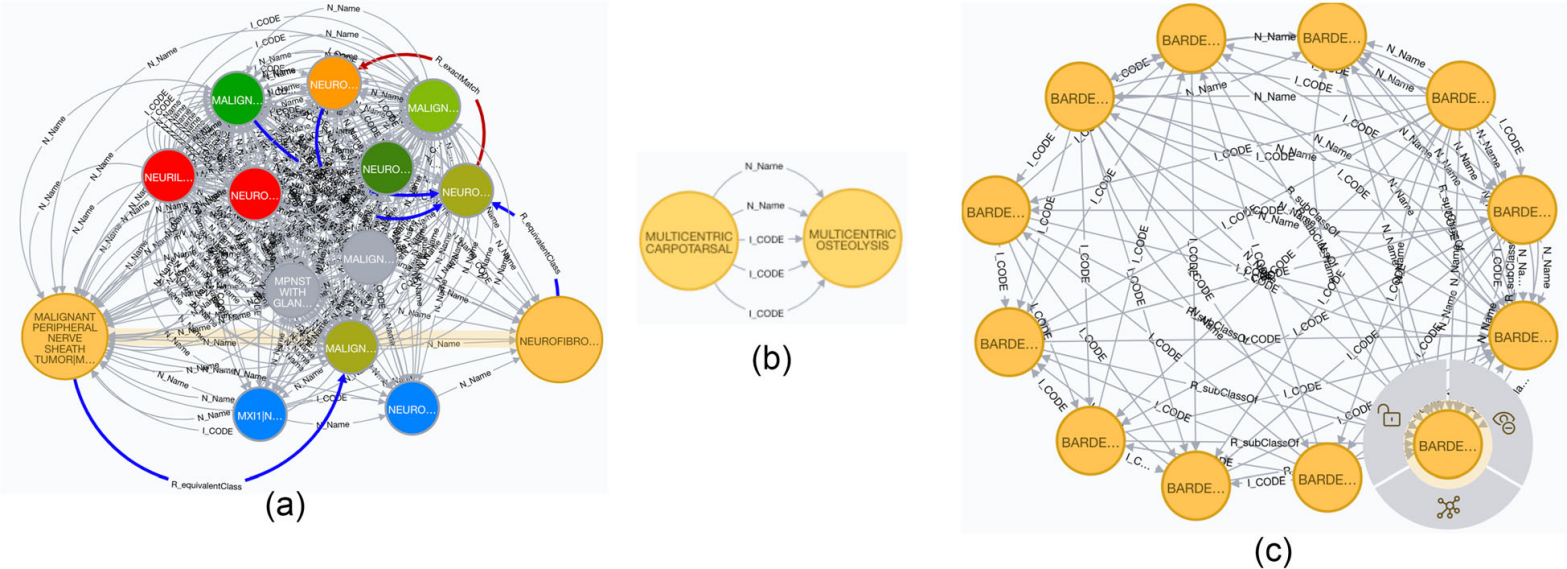
Mapping examples as guidance for data harmonization (yellow nodes denote GARD diseases; green nodes denote concepts from other resources, such as OMIM)

To improve nomenclature and data presentation/organization of GARD diseases, in this case study we attempted to systematically identify data harmonization rules by analyzing data mappings obtained from the knowledge graph. In other words, we aimed to identify any potential duplicate/similar GARD diseases via GARD internal mappings, by executing Cypher Query 3. Three examples shown in Fig. 2 are part of the result generated from this query.

**Cypher Query 3:** match p = (m:S_GARD)-[:I_CODE|:N_Name]-(n:S_GARD) return p limit 100.

**Description**: searching for 100 GARD diseases with the same disease name/code.

The first mapping example of “NEUROFIBROSARCOMA (GARD:0008211)” and “MALIGNANT PERIPHERAL NERVE SHEATH TUMOR (GARD:0010872)” is developed via one object predicate “N_Name” i.e., “NEUROFIBROSARCOMA”, the highlighted yellow edge shown in Fig. 2a. These two diseases are also mapped to multiple concepts from other resources, including OMIM in blue nodes, MONDO in purple nodes, MedGen in red nodes, etc. “MULTICENTRIC CARPOTARSAL OSTEOLYSIS SYNDROME (GARD:0013042)” and “MULTICENTRIC OSTEOLYSIS NEPHROPATHY (GARD:0003818)” are mapped via multiple mapping paths, “I_CODE” (i.e., “OMIM:16630”, “MONDO:0008152”) and “N_Name” (i.e., “MULTICENTRIC CARPO-TARSAL OSTEOLYSIS WITH OR WITHOUT NEPHROPATHY”, etc.), shown in Fig. 2b. These two examples illustrate some degree of disease similarity presenting among those diseases, which instructs us to define harmonization rules for filtering duplicate/similar GARD diseases with same concept name and/or identifier, or mapped to same concepts from other resources. The third example of 12 diseases, which are belonging to Bardet-Biedl syndrome family, are interlinking each other, shown in Fig. 2c. Apparently “Bardet-Biedl syndrome (GARD:0006866)” highlighted is a parent node of other eleven nodes. This example directs us to organize GARD disease with parent and child relationship. Before implementation of the harmonization rules, they will be reviewed and approved by our domain experts, including two co-authors with MD degree, AP is the director of the Office of Rare Disease Research (ORDR) at NCATS, ES is a Program Officer at ORDR at NCATS.

Besides data mappings via “I_CODE” and “N_Name”, there are other mapping strategies towards data harmonization by applying different types of data available from our Neo4j graph, such as, Genes, Proteins. In addition, we can employ network analysis with different topological properties, i.e., path length, to support data harmonization.

### Understanding pathogenesis of rare diseases

**Fig. 3:**
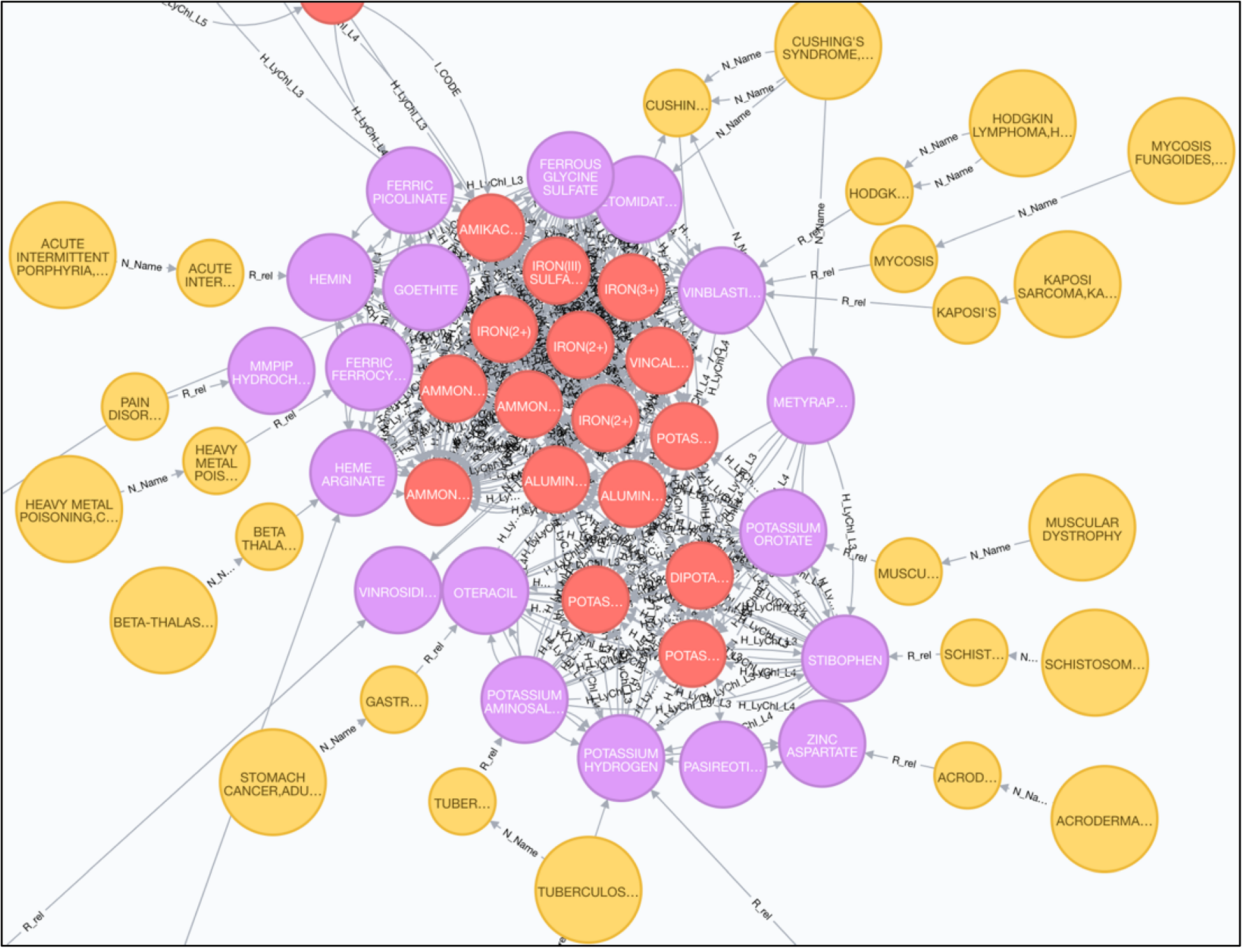
Demonstration of potential disease pathogenesis discovery for rare diseases (large yellow nodes denote GARD diseases; small yellow nodes denote conditions; purple nodes denote drugs; red nodes denote chemicals)

While researchers have made great progress in learning more about rare diseases and developing treatments for rare diseases, the exact cause of many rare diseases is still unknown, consequently most rare diseases still have no treatments. In this case study, we aimed to better understand underlying mechanisms among the rare diseases by exploring this integrative knowledge graph. A graph shown in Fig. 3, was generated to investigate pathogenesis among multiple GARD diseases including Cushing’s syndrome, meta-thalassemia, heavy metal poisoning, cystic fibrosis, acute intermittent porphyria, Hodgkin’s lymphoma, etc. These diseases are connected via multiple drugs, which share with common chemical elements, such as Iron, Aluminum, Potassium, etc. It illustrates possible underlying pathogenesis mechanism of those diseases is that they are associated with those common chemical elements. Literatures proved such findings, for example, Hodgkin’s lymphoma-anemia of chronic disease due to abnormalities in iron metabolism [26]. Iron deficiency is common in patients with cystic fibrosis [27]. Iron as a potential co-factor in the pathogenesis of Kaposi’s sarcoma [28]. Consequently the appropriate treatments can be determined for those diseases. Similar finding from the knowledge graph could assist further investigation on treatment discovery for other rare diseases.

## Discussion

In this study, we introduce an integrative knowledge graph by integrating GARD and other well-established biomedical datasets. We demonstrate four biomedical case studies with this knowledge graph in exploring the utilization of existing datasets in a disease-agnostic method and how that may further enhance scientific research on rare diseases. As a preliminary study, these initial results were generated with the goal of acting as a proof-of-concept to direct future development, and limitations from this study have provided key suggestions on continued refinements and next steps to improve this approach.

Since the inception of GARD in 2002, utilization volume of GARD increasingly grown over years, particularly in recent years, from about 270,000 average monthly users in 2016 to over 1.4 million average monthly users visited GARD, it is owing to comprehensive information about rare diseases provided by GARD. To further maximize wide use of GARD data, not only for its primary audience, including patients, their family and caregivers, health care professionals, but also for scientific researchers, will apparently accelerate the pace of rare disease diagnoses and scientific research in rare diseases. With this motivation, we developed this integrative knowledge graph by integrating GARD as well as other rare disease and biomedical data resources in structured and semantic form based on the predefined meta-ontology, which consists of our newly defined primary classes and object/data properties, and those adopted classes and properties from other resources, such as UMLS semantic types as class labels from MedGen, MeSH shown in Table 2. We acknowledge combination of these two lists of classes/properties might cause confusion for query composition, unless fully understanding the structure of individual datasets. As an ongoing effort, data will be continually collected and integrated into this knowledge graph, and the meta-ontology will be consequently expanded with more classes and predicates to be able to answer more complex scientific questions. Then we will merge those existing classes and object/data properties from other resources to our meta-ontology, for instance, the UMLS semantic type of “T109” (“Organic Chemical”) [25] from NCI Thesaurus will be merged to the class of “Chemical” defined in the meta-ontology.

There were two types of drug related datasets investigated in this study, orphan designated drugs that were organized from collected data submitted via new drug applications to the FDA, and a list of drug-disease-associations derived by manual curation from Inxight Drugs. These data were mapped to the GARD dataset through semi-automatic process. Though manual effort produced a high quality of mappings, this required substantial effort, time, and expertise from curators, from which the main option for scaling up a process to cover up to 7000 diseases, would be to increase the number of curators involved. In order to examine whether a comparable machine-based approach could either supplement or replace existing manual processes, we chose to utilize existing tools, including MetaMapLite [29], to trial a process for programmatically extracting disease terms from FDA orphan designations in free text. During this experiment, we encountered notable challenges in generating accurate annotations: 1) Abbreviations were frequently found in orphan designations text, which led to incorrect annotations, such as the use of “HD” for “Huntingtons Disease” or “SCD” for “sickle cell disease”; 2) Accommodating appropriate semantic types (ST) [25] for annotations proved difficult. If “dsyn: Disease or Syndrome” was selected as one ST for annotation, this could result in a missed annotation for the designation of “TREATMENT OF CONGENITAL LACTIC ACIDOSIS”, as “CONGENITAL LACTIC ACIDOSIS” associated with one ST of “cgab: Congenital Abnormality”. However, if additional ST were selected, unexpected annotations could also be generated. If “painful” was to be annotated as one ST of “sosy: Sign or Symptom”, this would lead to the mistaken association with “FOR RELIEF OF ALLODYNIA (PAINFUL HYPERSENSITIVITY), AND CHRONIC PAIN IN POSTHERPETIC NEURALGIA”; 3) A further process would be needed to prevent annotation of generic terms, such as “Disease”, “Disorder”, “Condition”, “Syndrome”, etc. A key lesson from this exercise was that an automated programmatic approach alone would create problematic annotations with a fair degree of variability due to off-target and false attributions. This led to our decision to implement a hybrid approach, combining an initial manual process supported by automated annotation, as our initial annotation goals had focused on structuring initial datasets to serve as a foundation for future data integration.

In using this hybrid approach, we generated mappings between FDA orphan designations and GARD diseases for which we found 2154 unique orphan designations were successfully mapped to 942 unique GARD diseases, as well as 3322 unique designated drugs that mapped to UNII. Not all orphan designations and designated drugs were mapped to GARD and UNII. The main reasons behind failed mapping were, 1) orphan designations that were for non-disease clinical sequalae, such as with toxic exposure in the case of “Treatment of cyanide poisoning” and “For use as an index of hepatic drug-metabolizing capacity”; 2) orphan designations that involved common disease or are too general, such as with “Treatment of patients with stage IIb, IIc, III, and IV melanoma”; 3) orphan designations for clinical symptoms or procedures, such as “Restore the structure and function of the esophagus subsequent to esophageal damage due to cancer, injury, or congenital abnormality”; or 4) a reflection of errors in the orphan designations themselves, such as incomplete phrases like “Treatment of pediatric patients 0 to”. Possible explanations for failed mapping between designated drugs and UNII include, 1) the lack of UNII codes available for those drugs; 2) the designated “Drug” involved a procedure rather than a therapeutic drug or biologic.

An overachieving goal in generating this integrative knowledge graph is to provide a publicly accessible database that integrates multiple well-established datasets for researchers of genetic and rare diseases in supporting scientific research and clinical decision making. An immediate use case of this comprehensive knowledge graph is with the aim of supporting re-development of GARD. Capturing this process and efficiencies enabled by it would allow us to promote a new model for generating consumer health information by focusing on sustainable, programmatic approaches that can both reduce redundancy in the data being collected and in the searching for and identification of reliable evidence sources. In addition to support scientific research, multiple extensions are proposed accordingly, 1) we will expand approved Orphan drugs to European Union by working with the Orphanet team; 2) we will assess impact/burden/progress of rare diseases from multiple different perspective, e.g., clinical trials, research funding; 3) we will apply advanced network analysis and computational techniques in Artificial Intelligence (AI) to support novel drug discovery and clinical decision making.

## Conclusion

Of the robust literature covering the development and utilization of biomedical knowledge graphs, most applications have focused on scientists and research professionals as well as with either common diseases or broad clinical and scientific disciplines. By successfully demonstrating preliminary results and retrieving biomedical insights across multiple rare disease information sources, we hope to apply the knowledge gathered from this process to begin expanding the available tools for rare disease researchers to investigate translational science concepts. If we can start to identify elements of a rare disease that impact the pace of scientific discovery, we may be able to leverage this knowledge to define diseases that are primed for therapeutic discovery or groups of diseases in need of platform approaches to advance beyond a research bottleneck. Ultimately, our principle aim with this knowledge graph is to provide patients with consumer health information that is more reflective of the current research data available on their disease and to scale this process up to include up to 7000 rare diseases. By continuing to integrate additional data sources into this knowledge graph and develop additional computational algorithms to derive meaning from their semantic relationships, we aim to enhance the ability of GARD’s website to help rare disease patients and caregivers in their decision-making and to reduce their diagnostic odyssey by supporting their ability to receive consumer health information that is reflective of the current scientific knowledge collected for their disease.

## Data Availability

All data can be accessed via https://disease.ncats.io

## Abbreviations

GARD: Genetic and Rare Diseases
OMIM®: Online Mendelian Inheritance in Man®
MONDO: Monarch Merged Disease Ontology
HPO: Human Phenotype Ontology
UNII: Unique Ingredient Identifier

## Footnotes

1 We restricted disease concepts with semantic type as “T047” from OMIM, to be mapped to GARD rare diseases by specifying “is_rare = true”.
